# Identification and characterization of bovine regulator of telomere length elongation helicase gene (*RTEL*): molecular cloning, expression distribution, splice variants and DNA methylation profile

**DOI:** 10.1186/1471-2199-8-18

**Published:** 2007-03-06

**Authors:** Zhuo Du, DingSheng Zhao, YongHui Zhao, ShaoHua Wang, Yu Gao, Ning Li

**Affiliations:** 1 State Key Laboratory for Agrobiotechnology, China Agricultural University, Beijing, 10094, China; 2 Institute of Space Medico-Engineering, Beijing, 100094, China; 3 Faculty of life science, Liaoning University, Shenyang,110036, China; 4 College of Animal Science and Technology, China Agricultural University, Beijing, 10094, China

## Abstract

**Background:**

The genetic basis of telomere length heterogeneity among mammalian species is still not well understood. Recently, a gene named regulator of telomere length elongation helicase (*RTEL*) was identified and predicted to be an essential participant in species-specific telomere length regulation in two murine species. To obtain broader insights into its structure and biological functions and to ascertain whether *RTEL *is also a candidate gene in the regulation of telomere length diversity in other mammalian species, data from other mammals may be helpful.

**Results:**

Here we report the cDNA cloning, genomic structure, chromosomal location, alternative splicing pattern, expression distribution and DNA methylation profile of the bovine homolog of *RTEL*. The longest transcript of bovine *RTEL *is 4440 nt, encompassing 24.8 kb of genomic sequence that was mapped to chromosome 13q2.2. It encodes a conserved helicase-like protein containing seven characterized helicase motifs in the first 750 aa and a PIP box in the C-terminus. Four splice variants were identified within the transcripts in both the coding and 5'-untranslated regions; Western blot revealed that the most abundant splice variant SV-1 was translated to a truncated isoform of RTEL. The different 5'UTRs imply alternative transcription start sites in the promoter; Bovine *RTEL *was transcribed at the blastocyst stage, and expression levels were highest in adult testis, liver and ovary. DNA methylation analysis of tissues that differed significantly in expression level indicated that relatively low DNA methylation is associated with higher expression.

**Conclusion:**

In this study, we have identified and characterized a bovine *RTEL *homolog and obtained basic information about it, including gene structure, expression distribution, splice variants and profile of DNA methylation around two putative transcription start sites. These data may be helpful for further comparative and functional analysis of *RTEL *in mammals.

## Background

Telomeres are specialized nucleoprotein complexes that form the natural ends of eukaryotic chromosomes and have important structural and protective roles. Cellular telomere length, which undergoes dynamic changes and is stringently regulated by a series of telomere-associated proteins, is fundamental to the understanding of cell growth and survival, replicative life-span and carcinogenesis [1-3]. In recent years, several factors other than the well-known telomerase regulation pathway have been shown to regulate telomere length considerably [4]: oxidative damage [5,6] or mutation of the telomeric DNA [7], high-order alternative structures, especially the G-quadruplex organized from guanine-rich regions [8,9], the T-loop structure of the telomere [10], illegitimate DNA recombinations and inappropriate repair of telomeric DNA all influence telomere length [4]. Recently, a novel gene named regulator of telomere length elongation helicase (*RTEL*), which encodes a helicase-like protein with several functional helicase domains/motifs, was shown to participate in telomere length regulation in two murine species [9,32]. By incorporating phenotypic data from *RTEL *knock-out ES cells and comparing the function of a nematode homolog gene named dog-1 (deletion of G tracts) [11], *RTEL *is predicted to unwind "harmful" structures that form in the G-rich region of genome, especially the telomeric DNA, in order to protect the telomere and influence the length balance [9]. As a potential G-quadruplex resolvase, RTEL plays an important role in telomere maintenance, embryonic development and survival in mice. At the same time, as a safeguard for the genome, it contributes to the maintenance of genetic stability by minimizing unexpected recombinations induced by G-quadruplex.

The genetic basis of telomere length heterogeneity among mammalian species is still largely unknown. *RTEL *was identified as an essential regulator of telomere length in mice, in order to obtain broader insights into its biological functions, information from other mammalian species may be helpful. It may be essential for regulating telomere length diversity in mammals in general, but little information has become available to date. Here we describe some basic information about a bovine *RTEL *homolog, including the genomic organization, cDNA sequence, expression distribution, forms of splice variant and status of DNA methylation, which may provide useful data for further comparative analysis. In addition, since *RTEL *could protect the telomere as well as prevent genome instability, both of which are critical for cell survival and longevity, *RTEL *may be a candidate for extending the replicative potential and life-span of cultured cells. Replicative senescence and the proliferative limit of *in vitro *cells is a major obstacle to the genetic manipulation of farm animals.

## Results

### Identification and characterization of bovine *RTEL*

Using bioinformatics tools, 11 overlapping EST sequences [GenBank: BE753036, DV890221, DV869581, DV923036, DV875515, BM031944, DV819111, DN514403, BI539060, CK947294, BE663182], which share significant homology to human and mouse *RTEL*, were identified and assembled. Together with genomic sequence information partly released by the Bovine Genome Project, this enabled us to obtain a 4.3 kb sequence encoding a protein with high similarity to the human and mouse homologs. Using two sets of primers, the coding region of bovine *RTEL *was amplified in two overlapping fragments (1.9 kb and 2.1 kb respectively) by RT-PCR. After sequencing and comparison with mouse and human *RTEL*, the coding region of bovine *RTEL *was identified. To determine the transcription start site and polyadenylation site, 5' and 3' RACE were performed: a 280 bp 3'RACE amplicon and two forms of 5'RACE amplicons (approximate 400 bp and 250 bp respectively) identical to the coding sequence were obtained by nested PCR. The full-length cDNA was then obtained by incorporating the coding and 5'/3'-untranslated regions. The longest transcript of bovine *RTEL *[GenBank: DQ323153] is 4440 nt, containing a 3801 nt major open reading frame flanked by a 257 nt (or alternatively 137 nt) 5'UTR and a 383 nt 3'UTR; the polyadenylation signal AAUAAA was identified 17 nt upstream of the poly(A) homopolymer. The nucleotide sequence of the coding region shows high identity with the human and mouse homologs: 70.9% with mouse *RTEL *[GenBank: NM_001001882], 76.6% with human NHL [33] (human homolog of *RTEL *[GenBank: NM_016434]; mouse and human show 77.1% identity with each other.

On the basis of the cDNA sequence and a short genomic contig sequence covering the 5'and 3' regions, the genomic sequence of bovine *RTEL *was obtained by five long-range PCRs and primer walking [GenBank: DQ323153]. The exon-intron boundaries were further identified by the cDNA and genomic DNA alignment program SIM4 [12]. The genomic sequence of bovine *RTEL *is distributed among 35 exons spanning 24.8 kb of genome, so it is more tightly organized than the human (about 40 kb) and mouse (about 37 kb) *RTEL *genes. All the exon-intron boundaries are consistent with the GT-AG splicing rule, and when all the exons are joined at the identified splice sites the product is identical to the bovine *RTEL *mRNA sequence. The exon/intron lengths and splicing donor/acceptor sequences are listed in table 1. Exonic sequences are in uppercase and intronic in lowercase. Because two forms of 5'UTR were identified, exon 1' and exon 2' represent the alternative first and second exons, respectively.

**Figure 1:**
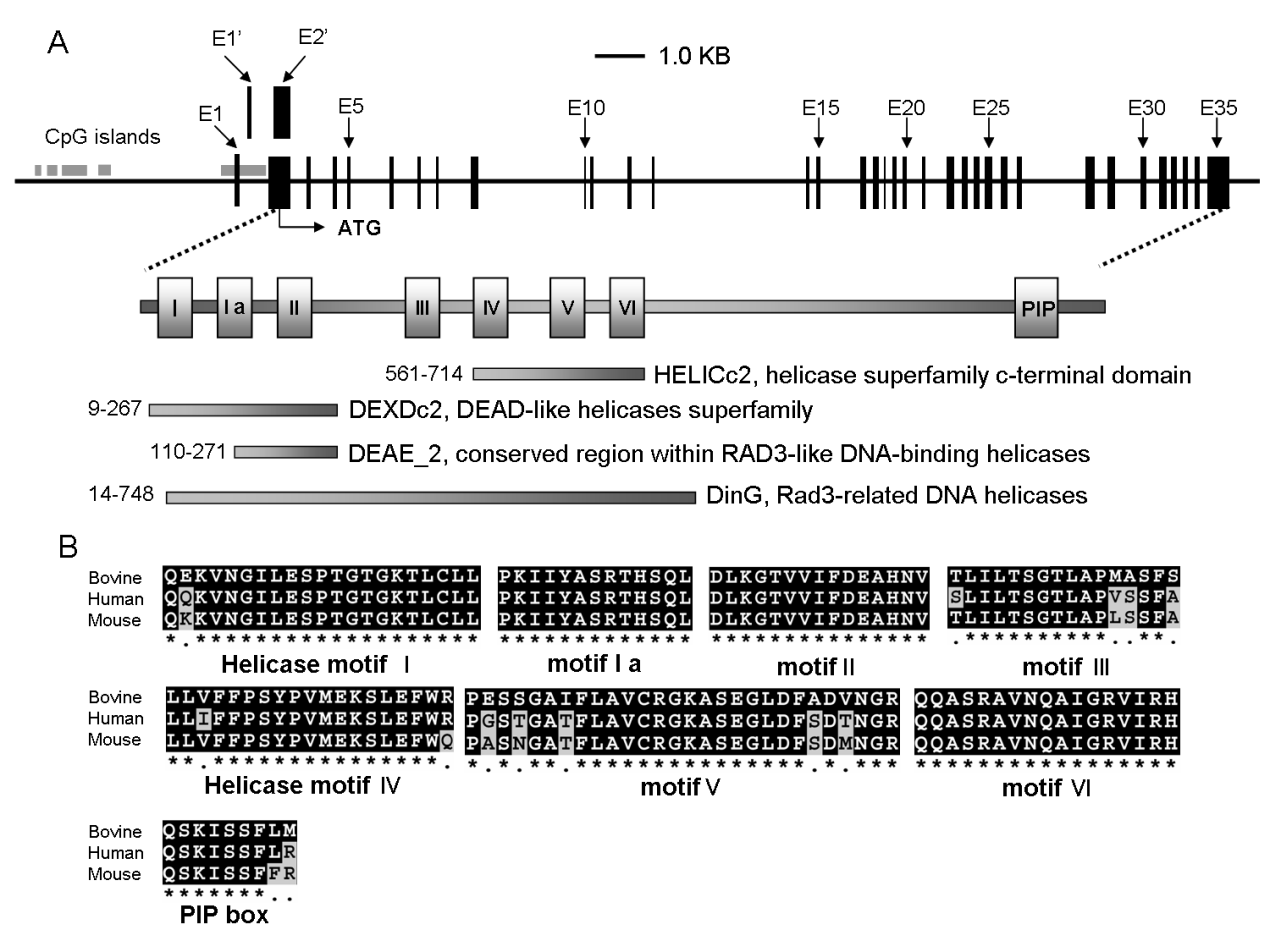
**Genomic organization, motif characterization and sequence comparison of bovine RTEL**. Bovine *RTEL *encodes a conserved helicase-like protein. A. Schematic representation of genomic organization and motif characterization of bovine RTEL. The genomic sequence of bovine *RTEL *is distributed among 35 exons spanning 24.8 kb of genomic DNA. The vertical lines represent exons (only partial exons are numbered); grey lines indicate CpG islands predicted in the putative promoter region. Because two forms of 5'UTR were identified, the exon 1' and exon 2' represent the alternative first and second exons respectively (see alternative splicing section for details). The deduced bovine RTEL protein contains three functional domains (DEXDc2, DEAE_2 and HELICc2) classified into the DinG, Rad3-related DNA helicases. The grey lines and related numbers below the schematic (not to scale) represent the position of each domain. The roman numbers specify seven characterized helicase motifs within the domains, and "PIP" indicates the PIP box identified near the C-terminus of RTEL protein. B. multiple alignments of deduced amino acids sequences in the eight identified motifs among three identified RTEL homologues. The consensus sequences of each motif are shown, identical and similar residues are shaded by black and grey respectively.

The complete coding region of the bovine *RTEL *transcript is predicted to encode a 1266 aa helicase-like protein with a calculated molecular mass of 139.8 kDa and a pI of 8.08. Motif analysis by searching the conserved domain database (rpsblast) of NCBI shows that as in human and mouse, the bovine RTEL protein is classified among the DinG, Rad3-related DNA helicases, which contain three functional domains (DEXDc2, DEAE_2 and HELICc2) with predicted transcription/DNA replication, recombination and repair functions (figure 1) [13]. In the conserved domains, seven conserved helicase motifs located in the first 750 aa were identified: helicase motifs I, Ia, II, III, IV, V and VI [14,15]. In addition, as in mouse and human, an eight amino acid motif (QXX(I/L/M)XXFF) PIP box was identified near the C-terminus of RTEL protein, which is predicted to be function as an interface interacting with proliferating cell nuclear antigen (PCNA) [16]. The representative domain/motif organization and multiple sequence alignment of bovine, human and mouse in the eight conserved motifs are shown in figure 1. In the helicase domain (first 750 aa), the amino acid sequence of bovine RTEL protein shares high identity and similarity with its mouse and human counterparts (86% and 91.9% with human NHL, 79.4% and 87.5% with mouse RTEL) and with other proteins with helicase activity.

In summary, these comparisons of the coding and deduced protein sequence, genomic organization and domain/motif structure indicate that we have identified the bovine homologue of *RTEL*.

### Chromosomal location of bovine *RTEL*

**Figure 2:**
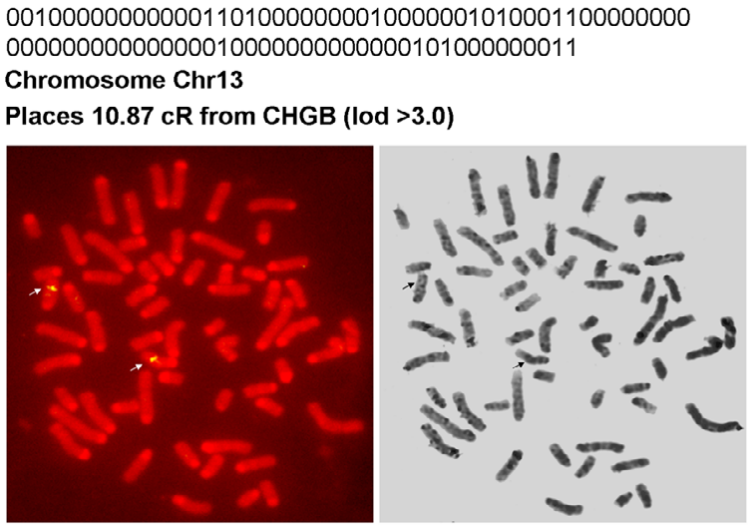
**Chromosomal location of bovine RTEL**. Bovine *RTEL *gene is mapped to chromosome 13 by radiation hybrid mapping (RH mapping) and fluorescence in situ hybridization (FISH). The numbers indicated the PCR results of RH mapping, 1 and 0 represent positive and negative signals respectively. *RTEL *was mapped to bovine chromosome 13, placed 10.87 cR from the CHGB mark (LOD>3.0). The FISH and G-banding results shown in the bottom further confirmed the RH mapping result, specific hybridization signals (arrows) were observed on homologous chromosomes 13q2.2 comparing to standard GTG-banded cattle karyotype.

To determine the chromosomal location of bovine *RTEL*, we carried out both radiation hybrid mapping (RH mapping) and fluorescence in situ hybridization (FISH). The amplification results of PCR screening of bovine 5 k (90 hybrids) radiation hybrid panels (00100000000000110100000000100000010100011000000000000000000000001000000000000 0101000000011) were submitted to the bovine radiation hybrid mapping service and analyzed by RHMAPPER-1.22. *RTEL *was mapped to bovine chromosome 13, placed 10.87 cR from the CHGB mark (LOD>3.0). In a previous study, homology was reported between bovine chromosome 13 and part of human chromosome 20 [17], where the human *RTEL *homolog NHL is located. Detailed comparative analysis based on the map position validated the RH mapping results. This shows that bovine chromosome 13 shares synteny with human chromosome 20 and mouse chromosome 2 in the DNAjc5 (a mark near CHGB) region, which contains *RTEL*. To confirm the chromosome position further, cytogenetic location was analyzed by FISH. As described in figure 2, four distinct hybridization signals were observed on two homologous chromosomes (arrows). After G-binding and comparison with a standard GTG-banded cattle karyotype according to size and distribution, we finally assigned bovine *RTEL *to chromosome 13q2.2. (When the study was performed, no location information was available in the bovine genome database. The chromosome location data are consistent with the Bovine Genome Project released recently.)

### Alternative splicing of bovine *RTEL*

**Figure 3:**
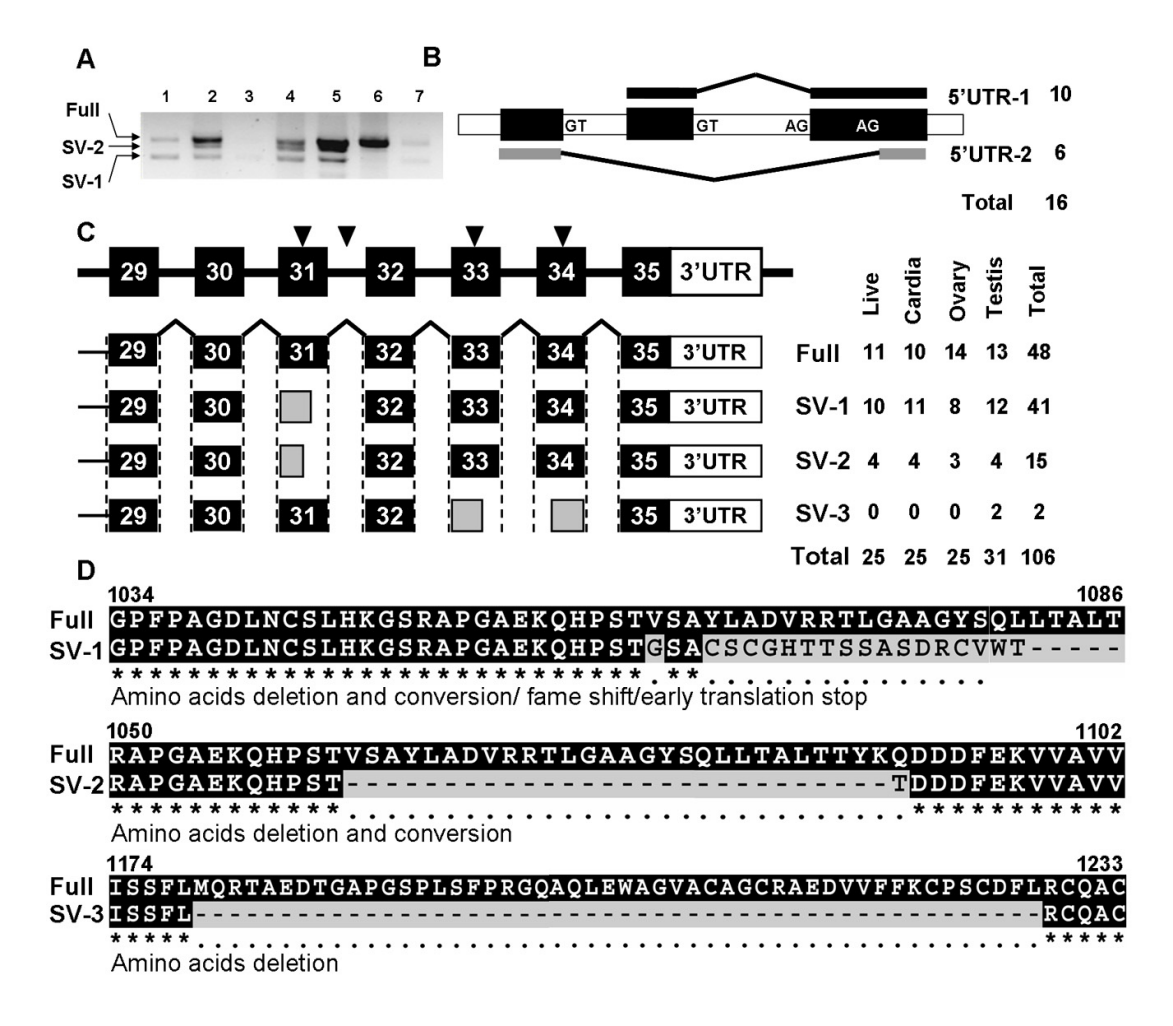
**Bovine RTEL is alternatively spliced in both coding sequence and 5' untranslated region (5'UTR)**. Three splice variants were identified in cDNA from multiple adult tissues, two 5'UTR isoforms were obtained by 5'RACE from testis. A. Agarose gel electropherograms of the splice variants from various tissues developed by RT-PCR, line 1 to 7 presents lung, liver, pancreas, cardia, testis, ovary and spleen respectively. The arrows indicate the splice variant correspond to each band. B. Two forms of 5'UTR. The rectangles indicate the genomic regions that identical to the 5'UTR (not to scale), putative exonic and intronic regions are indicated by filled and opened rectangles, GT-AG splicing sites were also shown. Skipping of full or partial exons is presented by brackets above or below according to different forms. Numbers on the right shows the frequency to observe a given 5'UTR isoforms. C. Splice variants in coding region. Number labelled boxes indicate the genomic regions identical to cDNA (exons 29 to 35). Regularly and alternatively spliced exons are shown by black and grey boxes respectively below. SV is short for splice variants and Full means the full-length transcript. Numbers on the right shows the times an splice variant presents in the sequenced *RTEL *transcripts from indicated tissues. D. Amino acids changes caused by alternative splicing. The alignment figures display the changes at the protein level of three identified splice variants (SV-1 to SV-3) compared to full-length protein, identical residues are shown in black background whereas changed are indicated by grey.

As alternative splicing is an efficient way to regulate gene function, we analyzed the forms and distribution pattern of splice variants in bovine *RTEL *transcripts from multiple adult tissues. On the basis of data about mouse *RTEL*, in which alternative splicing occurs only in the last four exons, primers covering exons 21 to 35 were used to analyze *RTEL *transcripts from various bovine tissues. A total of 106 clones of RT-PCR products from testis (31 clones), ovary, liver and spleen (25 for each) were sequenced. By comparison with the bovine genomic sequence, three coding region splice variants, mostly occurring in the last 6 exons (exons 30–35), were identified and characterized. Agarose gel electropherograms of the splice variants developed by RT-PCR are presented in figure 3A. Within the transcripts, 48 clones (45.3%) are full-length *RTEL *cDNA (named 'full'), 41 (37.7%) lack 166 bp from exon 31, indicating a selective 5' splice site (named 'SV-1' for splice variant-1), 15 (14.1%) lack 87 bp from exon 31(SV-2), and 2 (rare) clones from testis lack 150 bp from exons 33 and 34 indicating alternative 5' and 3' splice sites (SV-3). The patterns of the coding region splice variants and the relative tissue distributions are shown in figure 3C (GenBank accession nos. for the 3 splice variants are [GenBank: DQ420360, DQ420361 and DQ420362, respectively]).

The full-length *RTEL *cDNA and splice variant isoforms 1 and 2 are detected in high abundance in all the tissues examined; SV-3 is relative rare (2 out of 31) and is tissue-specific (detected only in testis). Since all three alternative splice variants are located in the coding region, there are considerable changes in the deduced protein sequences. The differences between the splice variants and wild type (the protein sequence deduced from the longest transcript) at the protein level are shown in figure 3D. For SV-1, the exon 31 truncation leads to a frame shift at codon 1063 and an early translation stop at codon 1084, generating a shorter peptide of 1083 aa lacking the PIP box. For SV-2, because the nucleotide lost is 3N, only 29 aa are lost followed by a Glu to Thr conversion without frame shift. Like SV-2, SV-3 lacks 50 aa and causes an Asp to Arg conversion.

To determine whether the first 21 exons also undergo alternative splicing, RT-PCRs were performed with primers covering exons 2 to 25 using pooled cDNA prepared from testis, ovary, liver and spleen. After 30 cDNA clones were sequenced, no alternatively spliced isoform was identified in this region. Therefore, like in mouse and human, alternative splicing of bovine *RTEL *is only detected in the last 5 exons.

Apart from the coding region variations, we also identified two forms of 5'UTR, named 5'UTR-1 and 5'UTR-2 [GenBank: DQ420364], by 5'RACE; 5'UTR-1 is the more commonly used form (10 of 16 clones). RT-PCRs were carried out using primers based on the differential region to verify that both forms of 5'UTR existed in the transcripts. The two forms were indeed confirmed by sequencing, at least in testis. Further analysis of the genomic sequence identical to the 5'UTR indicates that each form of 5'UTR is generated by selectively utilizing two of the three putative exonic regions, which is consistent with GT-AG rule: 5'UTR-1 selects the second and third exon-like regions, whereas 5'UTR-2 links the first and parts of the third exon-like regions by skipping the second one (figure 3B). Consequently, the 5'UTR sequences differ significantly, sharing only 55 nt in the region flanking the ATG site. Two mechanisms may be responsible for producing the different 5'UTRs: alternative splicing can explain the skipping of part of the third exonic region in 5'UTR-2 because an alternative AG acceptor site is observed in this region; whereas the selective utilization of the second exonic region in 5'UTR-1 may involve an alternative promoter or alternative transcription start site (TSS). Genes containing splice variants of this kind (figure 3B) usually have alternative promoters or, more exactly, alternative TSS. So on the basis of the different 5'UTRs, we presume that there are two TSSs for bovine *RTEL *transcription.

Overall, four splice variants were identified and characterized in bovine *RTEL *transcripts, three in the coding region and one in the 5'UTR. Among the coding region splice variants, SV-1, the most abundant isoform, encodes a prematurely truncated protein lacking the PIP box.

### Expression analysis of bovine RTEL

**Figure 4:**
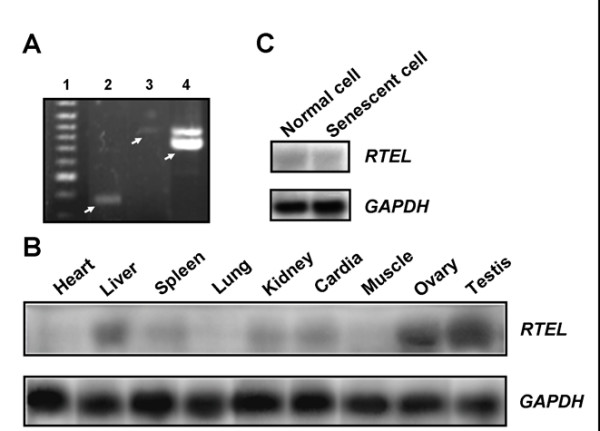
**Expression of bovine RTEL**. Expression of *RTEL *in blastocyst. Lines 1 to 4 indicates: marker, *GAPDH *amplicon, *RTEL *first round PCR and *RTEL *second round PCR. *GAPDH *serves as standard control, arrows highlight the amplification signals. B, C. *RTEL *expression in indicated adult tissues and cultured cells. A Two kb amplicon of bovine *RTEL *cDNA and 600 bp of *GAPDH *were labeled using [^32^P]dCTP to hybrid to normalized total RNA from multiple tissues and cells. The above panel shows ~4.5 kb hybridization signals of indicated tissues and cells, lower panel shows the ~1.3 kb *GAPDH *Northern blot signals as standard control.

The level of transcription in early developmental stages and the relative mRNA abundances in adult tissues and cultured cells were measured. Using the house-keeping gene *GAPDH *as internal control, RT-PCR analysis of nuclear transfer bovine embryos showed that bovine *RTEL *was expressed at the blastocyst stage. Further confirmation by a second PCR and sequencing showed that the common splice variant SV-1 was also present in the transcripts (Figure 4A). Then we analyzed the distribution of *RTEL *expression in adult tissues by using a [^32^P]dCTP-labeled two kb amplicon of *RTEL *cDNA and a 600 bp amplicon of *GAPDH *cDNA as probes hybridized to the size-fractionated total RNA; GAPDH served as control for RNA normalization. Northern blot data indicate that during embryo development and cell differentiation, the expression of bovine *RTEL *is restricted to germ cell lines (testis and ovary) and other metabolically active tissues with relatively high renewal potential including liver, spleen, kidney and cardia (Figure 4B). The size of the band was confirmed by comparing the relative positions of 18s and 28s ribosomal RNA. Among these *RTEL*-expressing tissues, testis shows the highest expression level; expression in liver and ovary is moderate; but in spleen, kidney and cardia the hybridizing signals are relatively weak. However, in other tissues including heart, lung and muscle, the expression level is below the detection threshold for Northern blot. Alternatively spliced transcripts of bovine *RTEL *cannot be visualized from the Northern results because the size-distinguishing capacity of the agarose gel is limited and the size difference between the splice variant and wild type transcripts is slight.

Since telomeres progressively shorten during cell division and senescence, we further compared the expression level of bovine RTEL between normal and senescent cultured cells. The expression data suggest that in cultured cells, the expression level of RTEL is very slight; and no significant difference was observed between normal and senescent cells. (figure 4C). The senescent cell phenotype was confirmed by a β-galactosidase activity assay (data not shown).

**Figure 5:**
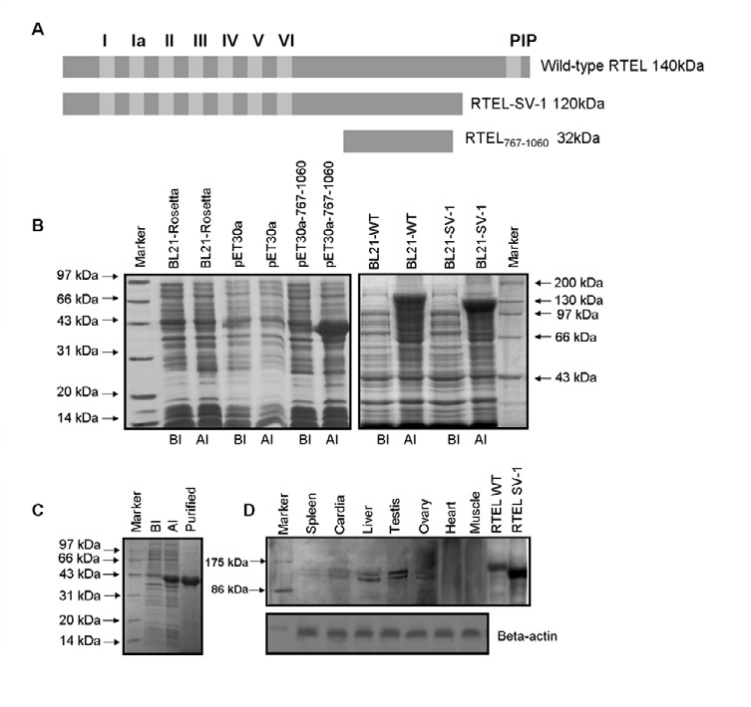
**Antiserum preparation and Western blot**. Three peptides, the wild-type RTEL protein, the RTEL isoform generated from splice variant 1 and RTEL767-1060 were over-expressed in E. coli. RTEL767-1060 was purified and used to prepare the antiserum for West analysis. (A) Schematic representation of the regions of RTEL that expressed in E. coli. BL21-Rosetta. RTEL-WT represents the complete coding region of RTEL; RTEL SV-1 represents the coding region of splice variants 1; RTEL767-1060 represents a shared coding regionin all potential isoform of RTEL. The roman numbers and 'PIP'specify the conserved helicase motifs and PIP box. (B). Over-expression of wild-type RTEL (WT), RTEL SV-1 (SV-1) and RTEL767-1060 (767–1060) in E. coli BL21-Rosetta induced by IPTG (1 mM). After inducement, the his-tagged recombinant proteins were over expressed. BI and AI listed below means 'before inducement' and 'after inducement' respectively. The empty pET30a-E. coli and wild-type BL21-Rosetta were used as negative controls. (C). Purification of his-tagged RTEL767-1060. RTEL767-1060 is a region shared by all potential RTEL isoforms and was used for antiserum preparation. The homogeneous of purified RTEL767-1060 is >95%. (D). Western analysis of RTEL expression. The lysates of E. coli cells that expressed wild-type RTEL and SV-1 RTEL were used as positive controls. Two bands were detected in the RTEL-expressing tissues, after comparing to the molecular marker and sizes of positive controls, the 140 kDa and the 120 kDa bands are corresponding to the wild-type RTEL and the SV-1 isoform of RTEL respectively.

Three splice variants in the coding region of *RTEL *(SV-1, SV-2 and SV-3) were identified in cDNA from various tissues, we further prepared antiserum against RTEL and performed Western blot to determine whether these splice variants, especially the highly expressed variant SV-1, were indeed translated. The predicted molecular weight of each protein variants of RTEL are 140 kDa for the wild-type RTEL protein; 119 kDa for RTEL SV-1; 137 kDa for RTEL SV-2 and 134 kDa for RTEL SV-3 respectively. In principle, besides the wild-type RTEL the isoform generated from SV-1 could be detected when using antibody that against the shared region of RTEL isoforms. We expressed the wild-type RTEL, RTEL splice variant 1 and a shared region of all potential RTEL proteins (amino acids 767 to 1060, RTEL_767–1060_) in *E. coli*. BL21-Rosetta. The over expressions of all recombinant proteins were induced by 0.1 M IPTG (figure 5AB). The his-tagged peptide RTEL_767–1060 _(approximate 37 kDa) was then purified and used to prepare rabbot antiserum. The homogeneous of purified RTEL_767–1060 _was >90% according to the result of SDS-PAGE (figure 5C). Total proteins of *E. coli *that expressed his-tagged wild-type RTEL and SV-1 RTEL (figure 5B) were used as positive controls for Western blot. The results of western blot (Figure 5D) shown that, as expected, the expression levels of RTEL protein are consisted with the mRNA abundance in the examined tissues (figure 4B) and there are two bands presented in the RTEL-expressing tissues. According to the molecular weight of the protein marker and compared the size with positive controls, the 140 kDa and 120 kDa band correspond to the wild-type RTEL protein and the RTEL splice variants 1 respectively (figure 5). Therefore, Western results indicated that the most aboundent splice variants are translated *in vivo*. However, it is difficult to determine whether other splice variants (SV-2 and SV-3) were translated from the Western blot because of the slight size difference between those variants and the wild-type RTEL protein.

### DNA methylation analysis

**Figure 6:**
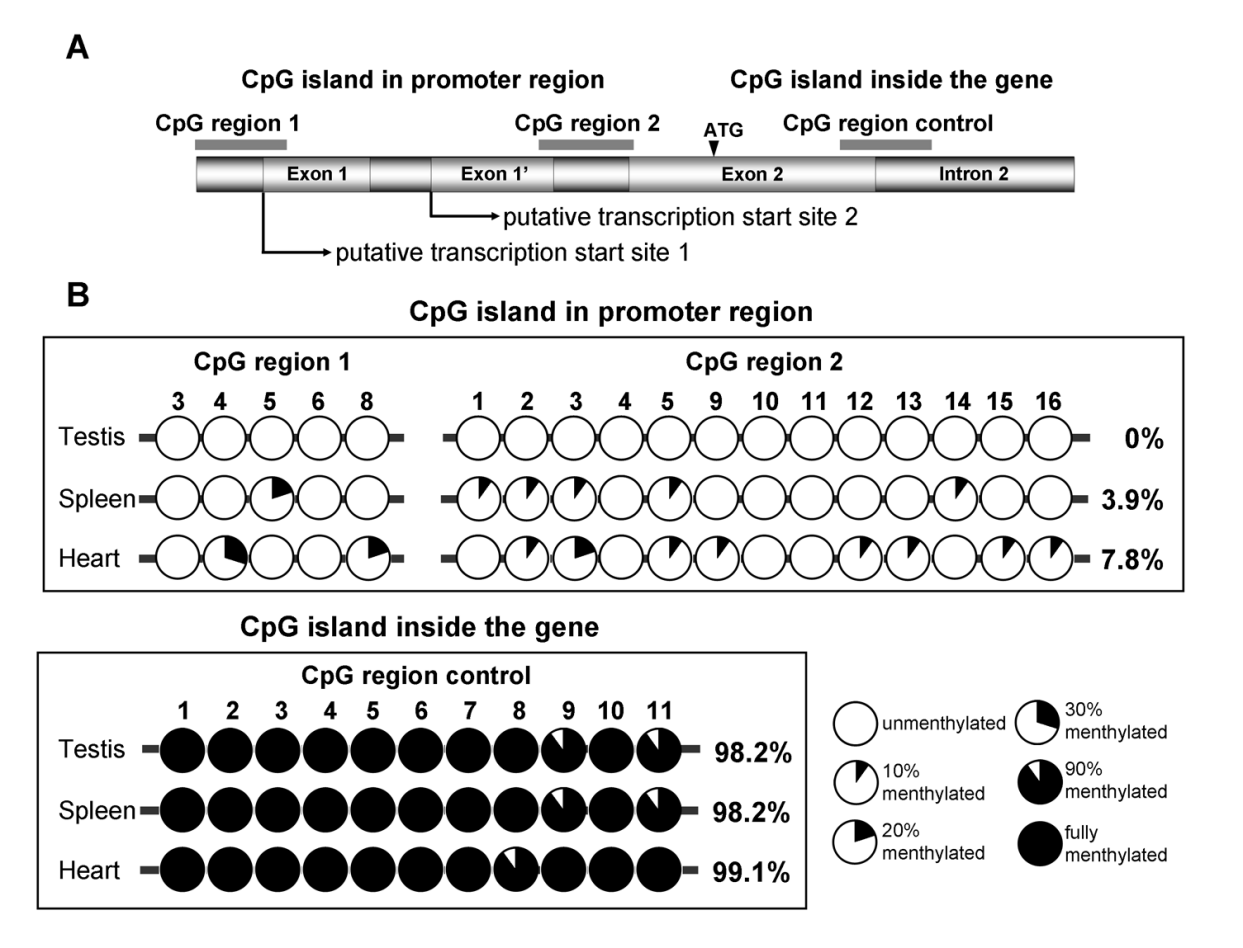
**DNA methylation profiles of three CpG regions in bovine *RTEL***. Three regions of CpG island were chosen for DNA methylation analysis, CpG region 1 (R1) and 2 (R2) are located in promoter region and CpG region control (RC) is in exon 2 and intron 2. A. Relative positions of three CpG regions be analyzed. Position of the islands are indicated by thick lines on the top, related genomic region and ATG site are indicated by boxes and arrow (not to scale), exon1 and exon1' mean alternative first exon and the arrows bellow indicate two putative transcription start sites. B. DNA methylation profile of the three selected regions. The cycles in a string mean the average methylation level of specific sites in each CpG region, the numbers above indicate the relative position of each CpG dinucleotides. Analyzed tissues (testis, spleen and heart) are indicated on the left. For the convenience to compare, CpG regions in the promoter regions (R1 and R2) and internal region are show separately. In each string, the open cycles indicate that the CpG sites are hypomethylated in all the clones that analyzed whereas closed cycles mean fully methylated CpG dinucleotides, other partially blacked cycles indicate different methylation level, details are shown in the right of the bottom. The average methylation level was calculated by sequencing ten individual clones of the PCRs. Besides the methylation level of each CpG sites, the percentages of DNA methylation in the indicated region of the CpG regions are also shown on the right (the data of R1 and R2 are incorporated to represent the methylation level of the promoter region). Only differential methylated CpG sites in three detected CpG regions are shown in the figure.

Analysis of the five kb genomic region flanking TSS of *RTEL *by the SIGNAL SCAN [18] and TFSEARCH [19] programs indicates that in the two kb region upstream of the TSS of *RTEL*, there is no TATA box or CAAT box, but a high density of CpG dinucleotides and several Sp1 sites were identified. In some promoters lacking TATA boxes, the Sp1 site is reported to be essential for efficient transcription [20], and in some TATA-less promoters there is always a high frequency of CpG dinucleotides, as in the telomerase catalytic reverse transcriptase subunit gene (*TERT*) [21,22]. Since the promoter region is rich in CpG dinucleotides, and six CpG islands were predicted by the methprimer program (Criteria used: island size > 100, GC Percent > 50.0, Obs/Exp > 0.6) [23], two regions in the largest CpG islands (859 bp) near the two putative transcription start sites (TSS) were chosen for analyzing the DNA methylation level and pattern by bisulfate sequencing. We name these two regions CpG regions 1 (R1) and 2 (R2). In addition, a putative CpG region containing 11 CpG dinucleotides covering exon 2 and intron 2 was selected as a control (CpG region control, RC) to represent the DNA methylation level inside *RTEL*. The relative positions of the 3 CpG regions are described in figure 6A: CpG R1 contains 19 CpG dinucleotides (CpGs) covering TSS 1, CpG R2 contains 16 CpGs placed 136 bp downstream of TSS 2, and CpG RC is placed inside the gene (exon 2 and intron 2). Three adult tissues with notably different expression levels including testis (with the highest expression level), spleen (medium) and heart (no detectable expression signal), were chosen for comparing the DNA methylation levels in these CpG regions. Genomic DNA for methylation analysis was extracted from the same tissue used for expression analysis in order to eliminate any error arising from different genetic backgrounds. In each tissue, 10 positive individual clones of the CpG region amplicons from three independent experiments were sequenced to give accurate measurements of methylation level and pattern. The DNA methylation profiles of the three indicated CpG regions in the selected tissues are presented in figure 6B. The bisulfite sequencing results (figure 6B) show that the DNA methylation level differs significantly between the promoter and internal (exon 2 and intron 2) regions. The methylation level increased from less than 8% in CpG R1 and R2 (in the promoter region) to more than 98% in CpG RC (inside the gene). As for the tissue-specificity of methylation, no difference in CpG RC (containing 110 CpG sites) was observed among the three tissues, which have significantly different expression levels. In heart, spleen and testis, the numbers of methylated cytosines are 109 (99.1%), 108 (98.2%) and 108 (98.2%) respectively. The detailed methylation levels of each CpG site are shown in figure 6B. However, in the specific regions of the CpG islands near the TSS (R1 and R2, containing 180 CpG sites), DNA methylation differed among the tissues. The numbers of methylcytosines in heart, spleen and testis are 14 (7.8%), 7 (3.9%) and 0 (0%), respectively. These methylation levels are negatively correlated with the *RTEL *expression levels in the tissues analyzed (figure 4B). In heart, which has the weakest expression, DNA methylation is relative high, whereas in testis, which has strong expression, the methylation level is low. This is consistent with the general notion that the DNA methylation level of specific CpG islands, which usually encompass the promoter and early exon regions, is negatively correlated with the gene expression level.

## Discussion

In this study, we have identified the bovine homolog of *RTEL *and provided some basic information including sequence, genomic organization, chromosomal location, expression pattern, splice variants and DNA methylation profile. From the mRNA and protein sequences, *RTEL *is to some extent conserved between bovine and mouse. Four splice variants were characterized in both the 5'UTR and the coding region, resulting in extensive changes in the protein sequence including amino acid substitutions and deletions, frame shifts and translation stops in the carboxy-terminus (figure 3). The high abundance of splice variants of *RTEL *in mouse, bovine and human indicates its potential functional importance. In mouse, splice variants may be an important clue to explain the telomere length difference between *Mus musculus *and *Mus spretus *[9]. As for the relative abundances of the splice variants, SV-1 and SV-2 are detected in the mRNA from all the tissues analyzed in relatively high abundance and show no tissue specificity, whereas SV-3 has low abundance and is only detected in testis. Its low abundance may result from tissue-specific alternative splicing. It is well known that many low-abundance splice variants may also contribute greatly to gene function because of the temporal specificity of splicing [24] or other unknown amplifying mechanism. At the protein level, all these splice variants produce novel C-terminus with low or no homology to the wild-type bovine *RTEL*, increasing the protein diversity. In addition, as alternative splicing occurs mainly in the last 5 exons, resulting in considerable amino acid changes in the region where few functional motifs/domains have been identified (750 aa-1266 aa), it also provides a good way to investigate the functions of this region. SV-1, where the PIP box is absent from the protein sequence, would be helpful for investigating the function of PIP and the functional relationship of RTEL and PCNA, and we have demonstrated that SV-1 indeed translated to a truncated protein by Western blot.

In mouse, five coding region splice variants were detected and identified in the last four exons (29 to 34) [9]. Some alternative splicing patterns are conserved between mouse and bovine: splice variant 4 in mouse resembles SV-1 and SV-2 in bovine. But our data show many differences from mouse: in the protein sequence, frame shift and early translation stop variants were observed; and one tissue-specific splice variant (SV-1) was identified (only detected in testis). Besides the coding region splice variants, we also identified two significantly different forms of 5'UTR (5'UTR-1 and 5'UTR-2). The data suggest that there are alternative transcription start sites in the promoter region. The organization and transcriptional regulation of *RTEL *is more complex than expected. Thisstudyprovides only a preliminary analysis of alternative splicing, It is still unclear how many of the splice variants we identified are functional and how many are aberrant ('noise'). Some splice variants may be by-products of false pre-mRNA splicing, so further analysis at the protein level and the biochemical properties of each variant is necessary. The functional significance of the different 5'UTR isoforms is also an interesting question for further investigation.

**Figure 7:**
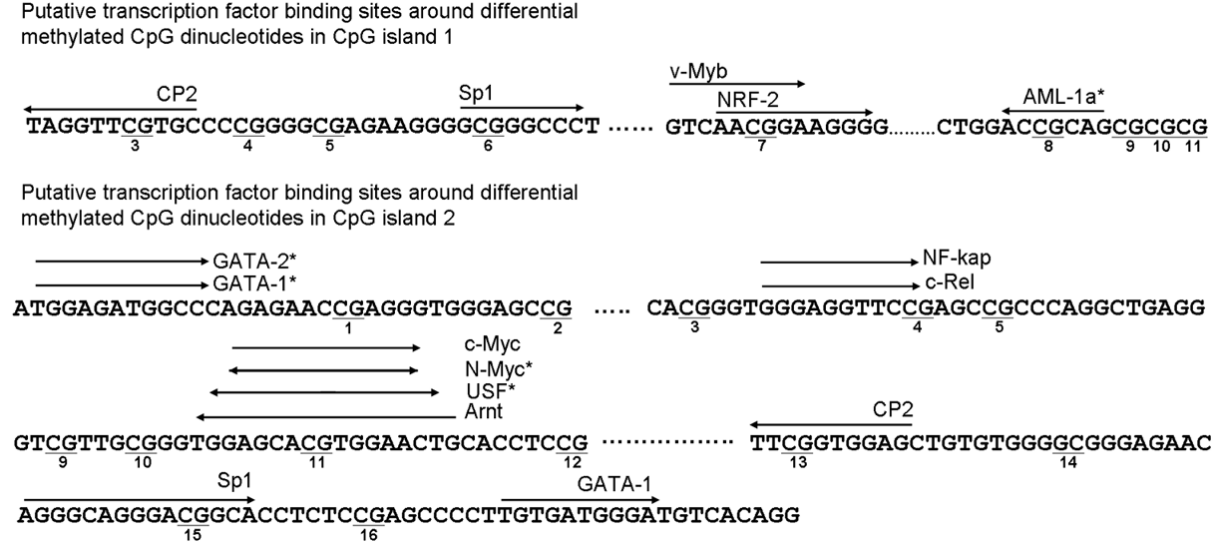
**Putative transcription factors (TFs) binding sites around differentially methylated CpG dinucleotides**. The sequence surrounding specific CpG sites are shown in the picture, differentially methylated and necessary neighbouring CpG dinucleotides are underline highlighted, the numbers below tags the relative position of each CpG sites in CpG island. The direction of the arrows above the sequence indicate the binding direction of TFs, arrow coved sequences are the binding consensus for each TFs tagged around the arrows. The binding pattern of TFs was analyzed by TFSEARCH programme Version 1.3 (Threshold score = 85.0), score>90.0 are asterisked.

To analyze the contribution of DNA methylation to the regulation of *RTEL *expression, we surveyed three CpG regions flaking bovine *RTEL*. The DNA methylation level differed between the promoter and internal regions, and tissue-specificity of the methylation level was found in the promoter CpG region (Figure 6B). A possible explanation of the cytosine methylation data is as follows: CpG RC, located inside the gene (exon 2 and intron 2), is not a "real" CpG island associated with gene expression because a functional CpG site usually encompasses the promoter and early exon regions. So the DNA methylation level in this region is very high (more than 98%) compared to regions 1 and 2, which are close to the TSS, and no differential DNA methylation was observed among tissues with significant expression differences (this is why we selected it as a control). In contrast, in CpG R1 and R2, which are located in the promoter region, considerably less DNA methylation (<10%) was observed, and tissue differences in methylation level were found. So compared to CpG RC, R1 and R2 may be important for transcriptional regulation. The difference in methylation may be relevant to the different gene expression levels in these tissues, higher methylation being correlated with lower expression. The slight difference in DNA methylation may be amplified by effects on the affinity for methyl-CpG binding proteins (MBDs), which influence the binding of specific transcription factors regulating gene expression. In figure 7, we show the putative transcription factor binding patterns associated with the differently methylated CpG dinucleotides, determined using the web tool TFSEARCH (with a threshold score of 85.0) [18]. However, the insignificant methylation differences (7.8%, 3.9% and 0% for heart, spleen and testis respectively) do not exclude the possibility that the low methylation level of the promoter region is merely an epigenetic permitting signal for gene transcription; the tissue-specific differences in expression pattern and level may be attributable to the transcription factors (TFs). An unmethylated or sparsely-methylated promoter region may be necessary but not sufficient for gene expression. Tissue or developmental differences in the expression patterns of some genes can be explained by differential DNA methylation [25]; for example, Fei Song et al. showed that tissue-specific differently-methylated regions (DMR) are common in the mouse genome, and most tissue-specific DMRs are associated with the expression pattern [26]. In contrast, Warnecke et al. found that the methylation level of the CpG island in the promoter region of the skeletal *α*-actin gene in mouse does not correlate with the tissue-specificity of expression [27]. In this study, only one of the six predicted CpG islands in putative promoter region of *RTEL *gene was inverstigated. The importance of the other islands needs further analysis and comparison.

Heterogeneity of telomere length is an important branch of the exciting field of telomere biology, many useful data have been provided by previous studies, paving the way for further revelations about the underlying mechanism. In the present study, we have identified and characterized the bovine homolog of *RTEL*, obtaining some basic information including expression pattern, alternative splicing and DNA methylation profile of the putative promoter region. All these data may be useful for further functional analysis and comparison of *RTEL *between bovine and other mammalian species. This would contribute to further understanding of the role of *RTEL *in regulating species-specific telomere length diversity. At the same time, in view of the capacity of *RTEL *to protect telomeres and maintain chromosome stability, this gene is also a candidate for extending the proliferative life span of cultured cells and preventing genomic instability, which currently vitiates the precise genetic manipulation of farm animals.

## Conclusion

In a previous study, *RTEL *was identified and functionally validated as an essential gene in the regulation of species-specific murine telomere length. However, little information is available about other mammalian species. In this article, we describe some basic information about bovine *RTEL*, with a view to providing useful data for further comparative and functional analysis. We have identified the bovine homolog and provided some comparative structural and functional information. Comparisons of the coding and deduced protein sequence, genomic organization and domain/motif structure indicate that *RTEL *is conserved between bovine and mouse. The expression patterns and modes of alternative splicing are also similar. The deduced promoter region of bovine *RTEL *lacks the typical TATA box and CAAT box, but presents a relatively high density of GC box and a high GC content, which is also seen in mouse. DNA methylation analysis indicates that in the tissues examined, ignoring differences in expression, the overall DNA methylation level is lower in the deduced promoter region; however, relatively low methylation was observed in tissues with high expression levels. To obtain broader insights into the biological functions of *RTEL *in mammals, our work may facilitate further understanding of the function of *RTEL *in bovines and elucidate whether *RTEL *is a candidate for regulating telomere length diversity in all mammalian species.

## Methods

### RNA preparing and gene cloning

Tissues (heart, liver, spleen, lung, kidney, cardia, muscle, ovary, and testis) were collected from adult cattle and were immediately stored in liquid nitrogen. All the experimental research on animals was follow internationally recognized guidelines. Total RNA was extracted from the above mentioned tissues using Trizol reagent (Invitrogen) according to recommendations of the manufacturer. Approximate 1 μg of total RNA was used for the first-strand cDNA synthesis (M-MLV reverse transcriptase, Promega) with d(T)18 primer. Two sets of primers (F1-5'CCC AAG AGT AGA CCA GTA TGC C, R1-5'ACA CGG ACA CTG AGA CAA TGC; and F2-5'CGG AGG TGA TGG AGG CTT TC, R2-5'AGT TCT GGG CAA GCA CAT TCC) were used to amplify bovine *RTEL *in two overlapping fragments. All the PCRs were performed in 25 μL containing 2 μL of cDNA, 2.5 μL of PCR buffer (Mg^2+ ^plus), 200 μM each dNTP mixture, 10 pM of each primer and 1 U Taq DNA polymerase. PCRs were run for 35 cycles of 94°C for 30s, 60°C for 30s and 72°C for 2.5 min, followed by incubation at 72°C for 10 min. The resulting PCR products were gel-purified and subsequently ligated into pMD18-T vector (TaKaRa) and sequenced. For each amplicon, three individual clones were sequenced on both strands. The primers used for cDNA cloning were also used for the detection of splice variants.

### Rapid amplification of cDNA 3' and 5' end

Total RNA from testis was used for 3' and 5'RACE using the 3'/5'-RACE kit (Invitrogen). For 3'RACE, first-strand cDNA synthesis was performed using the adapter primer (AP), then two rounds of 3'RACE-PCRs were performed using the abridged universal amplification primer (AUAP), and gene specific primer 1(GSP1) for first round; AUAP and GSP2 were used for the nested PCR. (3GSP1-5'GTG CCC TAC CTT GCC GAT GTC CG, 3GSP2-5'CCC TGA GAT GGC AGT GGG A). The PCRs was performed using the following conditions: 35 cycles of (94°C for 30s, 50°C for 30s and 72°C for 1 min) for first PCR; and 35 cycles of (94°C for 30s, 58°C for 30s and 72°C for 1 min) for nested PCR.

For 5'RACE, three nested GSP primers (5GSP1-5'CAC TGT TGT CTC CAG GCA GCT C, 5GSP2-5'GTA GCA GGC TGG GAC ATC TC, 5GSP3-5' CCT GCA GAC ACT CCA AAA CC) were designed on the basis of coding sequence. Primer 5GSP1 was used for first-strand cDNA synthesis at 42°C for 60 min; then cDNA was purified and added a homopolymere dC tail following the instructions of invitrogen 5' RACE kit. The following conditions were used for 5'RACE-PCRs: Abridged anchor primer (AAP) and 5GSP2 were used for first PCR in 35 cycles of (94°C for 30s, 50°C for 30s and 72°C for 1 min); AUAP and 5GSP3 were used for nested PCR in 30 cycles of (94°C for 30s, 58°C for 30s and 72°C for 45s). The PCR products were sequenced after gel purification and cloning.

### Amplification and sequencing of genomic region

Genomic DNA was extracted from blood using standard phenol protocol. To amplify RTEL, long-range PCRs (TaKaRa LA Taq™) were performed using five sets of primers complement to the exonic regions. All PCRs were performed by 35 cycles of 98°C for 10s and 68°C for 5 min, the resulting products were gel-purified and ligated into pMD18-T vector (TaKaRa), and sequenced by primer walking. The primer sets for long-range PCRs were as follow: (F1-5'ACA GGG AAG ACA CTG TGC CTT CTC, R1-5'CTG CTA CTT CAG GTT CCG AGA CAG; F2-5'TAC TAC CTG TCT CGG AAC CTG AAG, R2-5'GCT TGG TGA CAC CAC TGT TGT CTC; F3-5'GAG ACA ACA GTG GTG TCA CCA AGC, R3-5'TGT TCC AGA CAT CTG ATC GCT GAG; F4-5'AGG ACA GCT CAG CGA TCA GAT GTC, R4-5'CAA GGT CAT CGG AGC TCT TGT AGT; F5-5'GTC CCT CCC CAC CTA ACC TAA C R5-5'AGA GTC GAG TGG TCC CAG TTC G).

### Chromosomal location of bovine RTEL

Bovine 5 k (90 hybrids) radiation hybrid panel provided by Texas A&M University was used to determine the chromosomal location of *RTEL*. PCR screening was performed using the primer F-5'AAG TGA ACG GCA TTC TGG AG and R-5'CTG GGA CAT CTC CTT CCG GG. The results were submitted to BOVINE RADIATION HYBRID MAPPING and analyzed by RHMAPPER-1.22, The method of fluorescence in situ hybridization (FISH) was modified from Coppieters W et al. Briefly [28], Probe was labelled with biotin-14-dATP (Invitrogen) following the standard protocol, and coprecipitated with 50-fold excess of salmon sperm DNA. Hybridization was performed for 48 hours at 37°C in a humid chamber. Chromosomes were counterstained with 0.5 μg/mL propidium iodide. Images were taken with epifluorescence microscope equipped with a DP70 CCD camera (Olympus, Japan), and fluorescence signals were enhanced by software Video TesT-FISH (Video TesT Ltd, Russia, 2003). The chromosome slides were treated in 0.01% trypsin (Gibco) at 37°C for 50 seconds, and then stained with 0.04% Giemsa solution for G-binding.

### Embryo lysis and cDNA amplification

Four cloned blastocysts were used for preparing the template for expression analysis by protocol modified from those previously used [29]. PCRs were performed in standard condition: 35 cycles of 94°C for 30s, 58°C for 30s and 72°C1 min using primers F-5'CCA CAA GCA GCA GTT TGA GG and R-5'CAT GTT CAC TGG GGC TCT GG, the same primers were used for the second-round PCR under the same condition.

### Northern blot

Approximately 20 μg of total RNA extracted from adult tissues and fibroblast cells were size-fractioned in 1% formaldehyde-denatured agarose gel. Northern blot was performed following standard protocol, and α^32^-P labeled 2 kb amplicon of bovine *RTEL *cDNA and 600 bp region of housekeeping gene GAPDH were used as probes.

### Antiserum preparation and Western blot

cDNA region of wild-type RTEL, RTEL splice variant 1 and RTEL_767–1060 _were cloned into prokaryotic expression vector PET-30a (Novagen), all the inserts were verified by sequencing and transferred into BL21-Rosetta (DE3). The expressions of his-tagged recombinant proteins were induced by 0.1 M IPTG. The RTEL_767–1060 _peptide was purified by Ni-NTA resin (QIAGEN) and used to prepare rabbit antiserum. The homogeneous of purified RTEL_767–1060 _was >90% according to the result of SDS-PAGE. To prepare anti-RTEL_767–1060 _antiserum, two NZW Specific Pathogen-free (SPF) rabbits were immunized with the recombinant protein purified following standard procedure. Western blot was performed using anti-RTEL polyclonal antibody and anti-beta-actin antibody following the standard protocol.

### Bisulfate sequencing

Genomic DNA were modified by bisulfite based on previously described protocol [30,31]. -Twentieth of the treated template was used for PCRs in standard condition. To amplify each CpG region, we used 35 cycles of 94°C for 30s, 50°C for 30s and 72°C 30s for the first PCR; and 30 cycles of 94°C for 30s, 57°C for 30s and 72°C 30s for the hemi-nested PCR. Primers used for amplifications were listed as follows: For CpG region1: region1 F1 (5'AGT TGT TTG AGA GGG ATT TGG) and region1 R (5'TCT TCC CTA AAC TCT CTA CAC T) for first PCR, region1 F2 (5'GGG ATG ATT TTG AGG AGG GAG T) and region1R for hemi-nested PCR; the primers for CpG island 2 were: region2 F (5'GGA TGG AGA TGG TTT AGA GAA T) and region2 R1 (5'CAA ACC ACT TCA TCA ATT CCC) for first PCR, region2 F and region2 R2 (5'CCT ATA ACA TCC CAT CAC AA) for hemi-nested PCR; as for amplify CpG region control, primers regionC F1 (5'GGA GAGTT TTA TAG GTA TAG GGA AG) and regionC R1 (5'CCC ACC TCC CTA CCA AAA GTA AAA C) were used for first PCR and regionC F2 (5'AGG TAT AGG GAA GAT ATT GTG TTT T) and regionC R2 (5'AAA ACA TCA CCT AAA ACA TCT CCT T) for nested PCR. The resulting PCR products of each CpG islands was gel-purification and ligated into pMD18-T Vector (TaKaRa), and 10 random individual positive clones were sequenced.

## List of abbreviations

RTEL: Regulator of Telomere Length elongation helicase

UTR: Untranslated region

PCNA: Proliferating Cell Nuclear Antigen

RH mapping: Radiation Hybrid mapping

FISH: Fluorescence In Situ Hybridization

SV: Splice Variant

TSS: Transcription Start Site

## Authors' contributions

ZD participated in the study design, carried out the experiment including cDNA cloning, RH mapping, alternative splicing analysis, DNA methylation detection, prokaryotic expression data analysis and drafted the manuscript. DSZ participated in the study design and carried out the experiments including cDNA cloning, expression analysis, and helped to draft the manuscript. YHZ carried out BAC screen and FISH. SHW carried out bisulfate modification of genomic DNA and helped to DNA methylation analysis. YG carried out the prokaryotic expression and purification experiment. NL conceived the study, participated in the study design and revised the manuscript. All authors read and approved the final manuscript.

## Figures and Tables

**Table 1:** Genomic organization of bovine *RTEL*

Exon	Exon size (bp)	3'splice acceptor	5'splice donor	Intron size (bp)
1	97	/	GCCAG/gtcag	/
1'	82	/	TGGTG/gtgtg	/
2	457	tgtag/GATAT	CCCAG/gtgat	432
2'	353	cgcag/ACCCA	CCCAG/gtgat	872
3	94	ggcag/CCTGC	TACCG/gtggg	247
4	82	gccag/GCCCC	TGCAG/gtgcg	437
5	61	cccag/GTCCA	GGAAG/gtgag	195
6	76	cccag/AGAAG	CACAA/gtaaa	757
7	85	tccag/GCTCT	CCAAG/gtgag	444
8	66	tgtag/AGTCG	ATGTG/gtaag	272
9	154	cacag/GAGAA	GTCAG/gtgag	643
10	39	cccag/GGCTG	GAAGA/gtaag	2128
11	79	cacag/TGATT	GGGAG/gtgag	73
12	98	catag/CTACA	GGGAC/gtgag	654
13	56	cacag/GTGCC	TCCAG/gtagg	379
14	72	tccag/ATTGT	ACAAG/gtgag	3062
15	82	tccag/GTGCA	GCCAG/gtatg	186
16	133	tgcag/GGAAG	CAGAT/gtaag	760
17	114	tccag/CCCTT	AGACG/gtagg	120
18	41	tgcag/GTTTT	GCTGA/gtgag	82
19	86	tgcag/GCAAC	GGAGG/gtgcg	114
20	78	catag/GCCCG	CGGAG/gtncg	113
21	78	tccag/GTGAT	GGGAG/gtgag	350
22	147	tccag/GCTAG	GCCAG/gtgag	431
23	116	ttcag/TTCCT	CACAG/gtgca	105
24	124	tgcag/GTTTG	AGACT/gtgag	133
25	148	tctag/ATGCC	CACGG/gtgtg	88
26	158	tgcag/GCTCG	AGGAG/gtgag	145
27	96	ctcag/GCCTG	GCTCG/gtatg	145
28	199	tgcag/GAGGT	CCAAG/gtgcc	1246
29	141	cacag/GCTTC	CAGTG/gtgag	184
30	117	ttcag/GAAGG	CGCAG/gtact	527
31	235	atcag/GAGAC	CAGAG/gtgcc	158
32	146	tgcag/GTTCG	TCCAG/gtagg	75
33	117	tctag/GCCCC	GTGGG/gtagg	124
34	104	tgcag/CGGGT	TCCAG/gttgg	89

35	443	cacag/GTCTC	/	133

Exonic sequences are in uppercase, intronic sequences are in lowercase.

exon1' and 2' represent the alternative first and second exons deduced from 5'UTR-2

## References

[B1] de Lange T (2002). Protection of mammalian telomeres. Oncogene.

[B2] Smogorzewska A, de Lange T (2004). Regulation of telomerase by telomeric proteins. Annu Rev Biochem.

[B3] Blasco MA (2005). Telomeres and human disease: ageing, cancer and beyond. Nat Rev Genet.

[B4] Lansdorp PM (2005). Major cutbacks at chromosome ends. Trends Biochem Sci.

[B5] von Zglinicki T, Pilger R, Sitte N (2000). Accumulation of single-strand breaks is the major cause of telomere shortening in human fibroblasts. Free Radic Biol Med.

[B6] Tchirkov A, Lansdorp PM (2003). Role of oxidative stress in telomere shortening in cultured fibroblasts from normal individuals and patients with ataxia-telangiectasia. Hum Mol Genet.

[B7] Smith CD, Blackburn EH (1999). Uncapping and deregulation of telomeres lead to detrimental cellular consequences in yeast. J Cell Biol.

[B8] Crabbe L, Verdun RE, Haggblom CI, Karlseder J (2004). Defective telomere lagging strand synthesis in cells lacking WRN helicase activity. Science.

[B9] Ding H, Schertzer M, Wu X, Gertsenstein M, Selig S, Kammori M, Pourvali R, Poon S, Vulto I, Chavez E, Tam PP, Nagy A, Lansdorp PM (2004). Regulation of murine telomere length by Rtel: an essential gene encoding a helicase-like protein. Cell.

[B10] Wang RC, Smogorzewska A, de Lange T (2004). Homologous recombination generates T-loop-sized deletions at human telomeres. Cell.

[B11] Cheung I, Schertzer M, Rose A, Lansdorp PM (2002). Disruption of dog-1 in Caenorhabditis elegans triggers deletions upstream of guanine-rich DNA. Nat Genet.

[B12] Florea L, Hartzell G, Zhang Z, Rubin GM, Miller W (1998). A computer program for aligning a cDNA sequence with a genomic DNA sequence. Genome Res.

[B13] Marchler-Bauer A, Anderson JB, DeWeese-Scott C, Fedorova ND, Geer LY, He S, Hurwitz DI, Jackson JD, Jacobs AR, Lanczycki CJ, Liebert CA, Liu C, Madej T, Marchler GH, Mazumder R, Nikolskaya AN, Panchenko AR, Rao BS, Shoemaker BA, Simonyan V, Song JS, Thiessen PA, Vasudevan S, Wang Y, Yamashita RA, Yin JJ, Bryant SH (2003). CDD: a curated Entrez database of conserved domain alignments. Nucleic Acids Res.

[B14] Caruthers JM, McKay DB (2002). Helicase structure and mechanism. Curr Opin Struct Biol.

[B15] Tuteja N, Tuteja R (2004). Unraveling DNA helicases. Motif, structure, mechanism and function. Eur J Biochem.

[B16] Warbrick E (2000). The puzzle of PCNA's many partners. Bioessays.

[B17] Solinas-Toldo S, Lengauer C, Fries R (1995). Comparative genome map of human and cattle. Genomics.

[B18] Prestridge DS (1991). SIGNAL SCAN: a computer program that scans DNA sequences for eukaryotic transcriptional elements. Comput Appl Biosci.

[B19] Heinemeyer T, Wingender E, Reuter I, Hermjakob H, Kel AE, Kel OV, Ignatieva EV, Ananko EA, Podkolodnaya OA, Kolpakov FA, Podkolodny NL, Kolchanov NA (1998). Databases on transcriptional regulation: TRANSFAC, TRRD and COMPEL. Nucleic Acids Res.

[B20] Boisclair YR, Brown AL, Casola S, Rechler MM (1993). Three clustered Sp1 sites are required for efficient transcription of the TATA-less promoter of the gene for insulin-like growth factor-binding protein-2 from the rat. J Biol Chem.

[B21] Poole JC, Andrews LG, Tollefsbol TO (2001). Activity, function, and gene regulation of the catalytic subunit of telomerase (hTERT). Gene.

[B22] Cong YS, Wen J, Bacchetti S (1999). The human telomerase catalytic subunit hTERT: organization of the gene and characterization of the promoter. Hum Mol Genet.

[B23] Li LC, Dahiya R (2002). MethPrimer: designing primers for methylation PCRs. Bioinformatics.

[B24] Xing Y, Lee CJ (2005). Protein modularity of alternatively spliced exons is associated with tissue-specific regulation of alternative splicing. PLoS Genet.

[B25] Futscher BW, Oshiro MM, Wozniak RJ, Holtan N, Hanigan CL, Duan H, Domann FE (2002). Role for DNA methylation in the control of cell type specific maspin expression. Nat Genet.

[B26] Song F, Smith JF, Kimura MT, Morrow AD, Matsuyama T, Nagase H, Held WA (2005). Association of tissue-specific differentially methylated regions (TDMs) with differential gene expression. Proc Natl Acad Sci U S A.

[B27] Warnecke PM, Clark SJ (1999). DNA methylation profile of the mouse skeletal alpha-actin promoter during development and differentiation. Mol Cell Biol.

[B28] Coppieters W, Zijlstra C, Van de Weghe A, Bosma AA, Peelman L, van Zeveren A, Bouquet Y (1994). A porcine minisatellite located on chromosome 14q29. Mamm Genome.

[B29] Bloor DJ, Metcalfe AD, Rutherford A, Brison DR, Kimber SJ (2002). Expression of cell adhesion molecules during human preimplantation embryo development. Mol Hum Reprod.

[B30] Kerjean A, Dupont JM, Vasseur C, Le Tessier D, Cuisset L, Paldi A, Jouannet P, Jeanpierre M (2000). Establishment of the paternal methylation imprint of the human H19 and MEST/PEG1 genes during spermatogenesis. Hum Mol Genet.

[B31] Olek A, Oswald J, Walter J (1996). A modified and improved method for bisulphite based cytosine methylation analysis. Nucleic Acids Res.

[B32] Zhu L, Hathcock KS, Hande P, Lansdorp PM, Seldin MF, Hodes RJ (1998). Telomere length regulation in mice is linked to a novel chromosome locus. Proc Natl Acad Sci U S A.

[B33] Bai C, Connolly B, Metzker ML, Hilliard CA, Liu X, Sandig V, Soderman A, Galloway SM, Liu Q, Austin CP (2000). Overexpression of M68/DcR3 in human gastrointestinal tract tumors independent of gene amplification and its location in a four-gene cluster. Proc Natl Acad Sci U S A.

